# Assessment of Caspian Seal By-Catch in an Illegal Fishery Using an Interview-Based Approach

**DOI:** 10.1371/journal.pone.0067074

**Published:** 2013-06-26

**Authors:** Lilia Dmitrieva, Andrey A. Kondakov, Eugeny Oleynikov, Aidyn Kydyrmanov, Kobey Karamendin, Yesbol Kasimbekov, Mirgaliy Baimukanov, Susan Wilson, Simon J. Goodman

**Affiliations:** 1 School of Biology, University of Leeds, Leeds, United Kingdom; 2 Institute of Arid Zones, Southern Scientific Center of Russian Academy of Sciences, Rostov-on-Don, Russian Federation; 3 Institute of Microbiology and Virology, Almaty, Kazakhstan; 4 Institute of Hydrobiology and Ecology, Karasaysky Raion, Almaty, Kazakhstan; 5 Tara Seal Research Centre, Killyleagh, County Down, Northern Ireland, United Kingdom; Texas A&M University-Corpus Christi, United States of America

## Abstract

The Caspian seal (*Pusa caspica*) has declined by more than 90% since 1900 and is listed as endangered by IUCN. We made the first quantitative assessment of Caspian seal by-catch mortality in fisheries in the north Caspian Sea by conducting semi-structured interviews in fishing communities along the coasts of Russia (Kalmykia, Dagestan), Kazakhstan and Turkmenistan. We recorded a documented minimum by-catch of 1,215 seals in the survey sample, for the 2008–2009 fishing season, 93% of which occurred in illegal sturgeon fisheries. Due to the illegal nature of the fishery, accurately quantifying total fishing effort is problematic and the survey sample could reflect less than 10% of poaching activity in the north Caspian Sea. Therefore total annual by-catch may be significantly greater than the minimum documented by the survey. The presence of high by-catch rates was supported independently by evidence of net entanglement from seal carcasses, during a mass stranding on the Kazakh coast in May 2009, where 30 of 312 carcasses were entangled in large mesh sturgeon net remnants. The documented minimum by-catch may account for 5 to 19% of annual pup production. Sturgeon poaching therefore not only represents a serious threat to Caspian sturgeon populations, but may also be having broader impacts on the Caspian Sea ecosystem by contributing to a decline in one of the ecosystem’s key predators. This study demonstrates the utility of interview-based approaches in providing rapid assessments of by-catch in illegal small-scale fisheries, which are not amenable to study by other methods.

## Introduction

Fisheries by-catch of marine mammals, seabirds, and sea turtles is a global problem, and represents a critical threat for many long-lived, slow maturing species [1]; [2]; [3]. Seal-fisheries interaction problems occur everywhere fishing activity and seal habitat overlap, and by-catch is identified as an acute threat for some of the world’s most threatened pinniped species [4]; [5]; [6].

Evaluating by-catch can be challenging due to the logistical constraints on obtaining appropriate data on entanglement and mortality rates. For legitimate fisheries this is often achieved by independent observers recording by-catch from a known proportion of fishing effort in a fishery. However, this approach is labor and cost intensive, and is often supplemented by interviews with fishers. Obtaining information from small-scale fisheries can be more problematic, since these are often inaccessible to observers or are inadequately monitored, yet they comprise the majority of global fishing effort and may generate large by-catches of non-target vertebrates [7]. Illegal fisheries in particular, by their covert nature, generally cannot be observed directly by researchers and so are poorly studied, but they can be the source of significant by-catch. Quantifying by-catch in such fisheries to an approximate magnitude can provide vital information to inform policy decisions in the absence of other data. Direct questioning may be one of the most appropriate methods when resources are limited [8]. Here we show that an interview-based approach can yield data on by-catch in an illegal small-scale fishery over a short period of time, sufficient to estimate minimum annual by-catch rates, to identify high risk gear/location/season combinations, and to prioritize areas for further research.

At the end of the 19th century Caspian seals (*Pusa caspica*) were abundant with a population size of more than one million [9]; [10]. However, the population has declined by more than 90% since the beginning of 20th century, primarily due to unsustainable harvesting lasting until the 1990s [10]. The total Caspian seal population was estimated at approximately 104,000 in 2005, with an ongoing decline of 3–4% per year [10], [11]. The species is listed as endangered by the International Union for Conservation of Nature (IUCN) [12]. In 2012 the Caspian seal is still considered to be a ‘harvested’ species by the Russian Federation and faces a number of unresolved threats including habitat loss and degradation, disturbance from industrial development, potential decreased food availability due to over-fishing and invasive species, and fisheries by-catch.

From the early 1990s, following the collapse of the Soviet Union, state control of fisheries in the Caspian Sea have been very weak, resulting in a dramatic increase in illegal and unregulated fishing [13]. Extremely high economic values of sturgeon products, reaching up to US$10,000 per kg for beluga caviar, and unstable socio-economic conditions provided opportunities for the development of a substantial black-market. This illegal catch has driven catastrophic declines of all six Caspian sturgeon species reflected in the sharp decrease in sturgeon catch from about 30,000 tonnes annually in the 1900s down to less than 1,000 tonnes in 2007 [13], [14]. All five commercially important Caspian sturgeon species are now listed as critically endangered by IUCN [15]. The sturgeon gear used in illegal fisheries is indiscriminate, and therefore presents a potentially serious by-catch hazard to Caspian seals.

This study aims to quantify by-catch rates in the northern Caspian Sea to understand the potential contribution of by-catch to the ongoing Caspian seal population decline. The results are also essential for developing and implementing effective conservation strategies for Caspian seals, and for providing evidence to incorporate these measures into official policy in the Caspian littoral states, since no quantitative data on the scale of the catch are currently available, and the governments of the Caspian states have not formally recognized the issue.

## Materials and Methods

### Interview Survey Design, Cultural Context, and Study Area

We used an interview-based method commonly employed for by-catch studies [4], [7], [16], which was considered to be the most appropriate approach for the Caspian Sea where fishing is poorly regulated and recorded.

Dedicated by-catch interview surveys were conducted in the main fishing settlements along the Russian shoreline of the Caspian Sea (in the Republics of Kalmykia and Dagestan, 1–11 September 2009) and in Kazakhstan, (2–8 October 2009). Further interviews in Kazakhstan, 19–27 April 2009, and in Turkmenistan, 25–26 September 2009 were carried out during field trips dedicated to seal monitoring and tagging. Semi-structured individual and group interviews were conducted with fishermen, local representatives of the Federal Agency for Fisheries, Border Guard Service and State Small Boat Inspectorate in each country. Participants were identified through (non-random) opportunistic and ‘snowball’ sampling which is widely used in sociological research of illegal activities [17], [18]. The interview questionnaire and further details of the interview protocol are given in the supplementary information (Section S1 in File S1).

Approximately 100 people contributed to a total of 78 interviews (including 18 group interviews) in 31 settlements (54 interviews from Kalmykia and Dagestan, 22 from Kazakhstan and 2 from Turkmenistan). The interviews yielded 102 reports of independent by-catch events or absence of by-catch. Of these reports, 65 (37 and 28 boats engaged in sturgeon and ordinary fishing respectively) gave quantitative information about incidental by-catch while one reported deliberate killing of seals.

### Data Analysis

#### Estimate of total minimum by-catch

Most fishermen did not keep personal records, and therefore were unable to give detailed quantitative accounts of their individual total fishing effort, the number of trips or net sets, throughout the year, which prevented the calculation of an accurate scaling factor for average by-catch rates. As the most conservative alternative we therefore asked fishermen to focus on reporting the maximum number of seals encountered in a single by-catch incident during the September 2008-September 2009 fishing season. The sum of these reports across boats was taken as the minimum documented by-catch, allowing that there would be a variable amount of additional by-catch for each boat dependent on the frequency and mode of their fishing operations. We recorded the number of seals per net, per set or per group of nets, together with the total length of nets where provided. However, responses were often variable and it was not always possible to get fishermen to express by-catch in a consistent way. For example if a fisherman reported that a *minimum* of 2 seals were caught per every 100 m group of nets, in a 1 km set of nets, we recorded this as 20 seals for the 1 km set. If it was reported as *about* 2 (or 1–3) seals caught per 100 m group of nets for a 1 km net set, we recorded this as 10 seals per 1 km, allowing that not every 100 m group may contain seals. Some fishermen expressed by-catch in time periods e.g. 5 seals a week during one fishing month - in this case we recorded this as 20 seals. In order to avoid over-estimating by-catch rate, the number of seals per set was counted once even when it was reported to occur multiple times using qualitative phrasing such as “sometimes”, “often” or “regularly”. A total of 14 out of 65 reports (including 11 of 60 reports for 2008–2009) required adjustment in one of these ways. Results based on data with and without adjustment are provided.

Qualitative statements such as “some”, “few” or “many” animals by-caught were excluded from quantitative analysis. However, we included them in the total number of by-catch events, i.e. each of these statements was counted as one incidence of by-catch. Some quantitative reports referred to earlier years: 2002 (1 report), 2006 (3 reports) and 2007 (2 reports). These data were excluded from the minimum number of seals by-caught for 2008–2009, but were used for estimating by-catch according to type of gear and fishing area.

**Figure 1:**
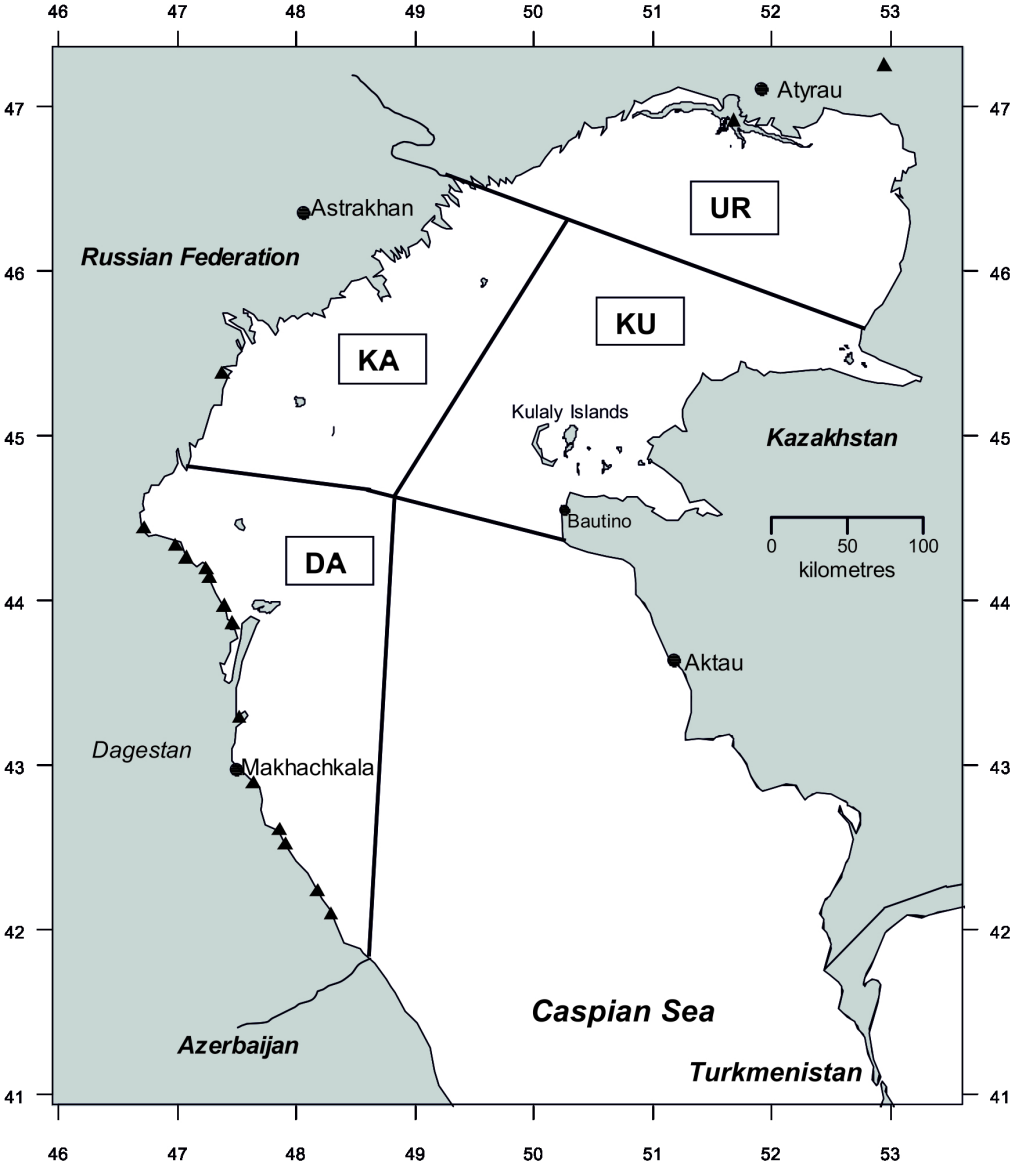
Map of northern Caspian showing settlements in which interviews were conducted (triangles), by-catch sectors. (UR - Ural; KU - Kulaly; KA – Kalmykia; DA – Dagestan; delimited by lines).

Quantitative by-catch data were assessed to identify high risk gear, locations and seasons. Gear type was divided into two groups: “ordinary” fishing nets (gillnets with mesh size 30–90 mm and fyke nets) which target a range of small non-sturgeon fish species, and illegal sturgeon fishing gear (gillnets with mesh size 100–250 mm and hook-lines) used by poachers and aimed at catching sturgeon for meat and caviar. Interview responses fell into three categories according to season reported: Winter-Spring (February-April); Autumn (September-November) and Autumn-Spring (fishing in either September-November, or after ice melt, but exact period not given). Reports without season specified, or reported as Autumn-Spring by fishers, were excluded from the season comparison analysis as uninformative. The fishing area was divided into four geographic sectors: Ural – the northern-most part of Kazakhstan waters; Kulaly - Kazakhstan waters including Kulaly archipelago; Kalmykia – northern part of Russian waters; and Dagestan – southern part of Russian territorial waters (Fig. 1). Turkmenistan was not allocated a sector due to the small number of interviews for this area. Data were summarised by taking the total by-catch, mean by-catch rate (seals/boat/year), standard deviation, and range. Means were compared via Wilcoxon rank sum tests with continuity correction. Due to uncertainty over the total north Caspian Sea fishing effort, and nonrandom sampling, it was considered inappropriate to extrapolate documented by-catch to a fleet-wide estimate based on the available data.

#### Post-mortem investigation of stranded carcasses in kazakhstan

In May 2009 a mass stranding of Caspian seals occurred along the north-eastern (Mangistau) coast of Kazakhstan (see Fig. 1). The area was surveyed by boat between 15 and 18 May. Carcasses were examined visually for the presence of entangling net fragments, or physical trauma consistent with net entanglement or fisheries interaction, e.g. lesions of dermal tissues around the body caused by embedded net ropes.

### Research Ethics

The interview procedures, interviewee data handling and data analysis methods were reviewed and approved by University of Leeds, Faculty of Biological Sciences Research Ethics Committee (review reference: BIOSCI 12-012). The dead animals were from fisheries by-catch, reported by fishermen to be from accidental drowning in fishing nets, an event that was independent of the study. No samples were taken from animals for any of the analyses presented in this study.

## Results

### Fishing Activity in the Study Area

Fishing in the study area is conducted by small open motor boats between 3 and 10 m in length powered by outboard engines ranging from 50 hp for inshore activities, up to 1000 hp for boats operating long distances offshore. Fishermen operate from the border of the reed zones up to 300–400 km from their home port. Dagestanian fishermen reported they regularly enter Kazakhstani territory (illegally) for sturgeon.

Boats are owned by private individuals working for official fishing cooperatives or illegally. Gillnets were the most common type of gear both in official and illegal fisheries, reported in more than 90% of all reports (Table 1), with mesh-sizes ranging from 30 mm to 250 mm depending on target fish species. The main non-sturgeon target species are: herring (*Alosa kessleri)*, sazan *(Cyprinus carpio* **)**, vobla (*Rutilus rutilus caspicus)*, kefal *(Liza aurata),* kutum *(Rutilus frisii kutum)*, bream *(Abramis brama),* cat-fish *(Silurus glanis)*, shemaya *(Chalcalburnus chalcoides),* inconnu (*Stenodus* leucichthys), and salmon (*Salmo trutta caspius*). Gillnets used for these fish normally have mesh of 30–90 mm. One set of nets deployed from one boat typically includes 5–7 individual nets each approximately 25 m long, giving a total length of 125–175 m. Fyke nets are commonly used in coastal fisheries.

**Table 1:** Results of fishermen interviews on seal by-catch in 2009 including reports of usage of different types of fishing gear, by-catch and hunting, seal depredation and usage of seal products.

Reports	Number of reports		
	Ordinary fishing net(<90 mm mesh)	Sturgeon fishing nets (100 mmand more mesh) andhook-lines	Total
All reports (number of statements)	40	53	93 (+9 where statement did not include net type)
Reports of gillnets usage (number of statements)	38 (95%)	48 (91%)	86
Reports of seal depredation on fisheries (number of statements)	26 (65%)	0	26
Reports that seals are detrimental to fishing activities (number of statements)	1 (3%)	0	1
Reports of seal by-catch cases (number of statements)	13 (33%)	49 (92%)	62 (+2 where statement did not include net type)
Seals reported by-caught(number of seals)	2008–2009: 55 Other years: 0Total: 55	2008–2009: 798 Other years: 168Total: 962	2008–2009: 853 Other years:168Total: 1017
Adjusted number of seals by-caught	2008–2009: 79 Other years: 0.Total: 79	2008–2009: 1215 Other years: 215Total: 1431	2008–2009:1294 Other years: 215Total: 1510
Reports of seal skin use (number of statements)	39 (incl. 32 cases related to 2008–2009 fishing year)		
Reports of seal blubber use (number of statements)	11 (incl. 10 cases related to 2008–2009 fishing year)		
Reports of illegal seals hunting(number of statements)	12 (incl. 9 cases related to 2008–2009 fishing year)		

Sea fishing for sturgeon species, which is illegal in the northern Caspian Sea, is normally conducted with nets of 110–250 mm mesh-size, set in water depths from 1 to 30 m. Typically, one set of sturgeon nets can include 5–20 groups (gangs) of linked nets of 100–200 m length each, giving 1–4 km in total for each set. Sturgeon fishermen also use hook-lines which can be up to 2,000 m in length and set in different water depths. Hook-lines comprise about 1,000 baited or self-catching unbaited hooks which are attached to the main line using 30–40 cm lengths of line, which entangle sturgeon if they pass through the line.

### Seal Interactions with Fisheries

Fishermen did not regard depredation of commercial fisheries by seals as a significant problem (Table 1), with only one report that seals were detrimental to fishing activities. However seals were commonly reported to damage some fish (65% of reports), particularly herring, inconnu and shemaya, in nets (Table 1).

### By-catch Rates in Relation to Type of Fishing Gear, Season and Area

13 of 40 (33%) reports from “ordinary” gillnet use reported incidental seal by-catch, whereas no by-catch was reported from fyke nets. Seals were regularly by-caught in gear used in sturgeon fishing (49 of 53 (93%) reports of sturgeon fishing; see Table 1). Mass entanglements of pinnipeds are rare in by-catch literature, but Caspian fishermen often reported large numbers of seals (>20) entangled together in a single sturgeon net (see Figure S1 in File S1). Average minimum rates were 1.84 seals/boat/year (ranging from 0 to 25, standard deviation - SD = 5.09) for “ordinary” gear, and 34.19 seals/boat/year (ranging from 2 to 125, SD = 32.62) for “sturgeon” gear (p<0.001, W = 44.5, Wilcoxon rank sum test with continuity correction).

The total number of seals by-caught varied between season and area (Table 2); with most by-catch reported between the breeding season in February when moulted pups are dispersing from the melting ice and the end of April when seals are dispersing from molting sites. The highest by-catch numbers (326 seals from 6 reports relating to the Kulaly sector) were reported by Russian poachers operating in Kazakh waters in February-April, citing cases of up to 100 seals caught per fishing trip, typically in sets of ∼2 km of nets. Partitioning the data by area yielded small samples that did not allow meaningful statistical comparison of by-catch rates since their distributions did not conform to underlying assumptions for either parametric or non-parametric tests (data not shown). By-catch rates in winter-spring were higher than in autumn, although the differences were not statistically significant (p>0.05, Wilcoxon rank sum test with continuity correction; see table 3). The comparison is likely to have low power due to reduction in sample sizes when partitioning the data by season. No fishers reported by-catch of seals during summer months.

**Table 2:** Breakdown of minimum reported by-catch in 2008–2009 by area and season.

Reported by-catch 2008–2009						
Area	N	Winter-Spring	Spring/Autumn	Autumn	Not specified	Total
**KU**	6	240	20	60	6	326
**UR**	8	100	0	33	3	136
**KA**	13	0	0	31	3	34
**DA**	31	215	52	38	46	351
**Turkmenistan**	2	6	0	0	0	6
**Total**	**60**	**561**	**72**	**162**	**58**	**853**

N – number of reports with quantitative data; Winter-Spring: February-April; Autumn: September-November; autumn-spring: fishing in either September-November, or after ice melt, but exact period not given; Not specified: no season information given by interviewee.

**Table 3:** Comparison of by-catch rates (seals/boat/season) among seasons.

Gear type	Season	N	Mean	Median	Range	SD
**All gear**	Winter-Spring	12	33.0	28.5	0–100	32.3
	Autumn	17	21.7	6.0	0–125	37.2
	*Wilcoxon rank sum test*	W = 75.5, p-value = 0.2481				
**Sturgeon gear only**	Winter-Spring	9	44.0	34.0	10–100	29.8
	Autumn	11	31.7	8.0	3–125	43.5
	*Wilcoxon rank sum test*	W = 27, p-value = 0.09414				

N – number of reports with quantitative data; Winter-Spring: February-April; Autumn: September-November; Mean, Median and SD (standard deviation), refer to seals/boat/season; Range refers to reported minimum by-catch in the sample.

The total minimum number of seals reported to be by-caught across all sectors (Table 1) was 853 seals for 2008–2009, with 798 seals from sturgeon fishing gear. Adjusting reports for multiple sturgeon gear sets yields a total minimum estimate of 1,215 seals by-caught from 31 sturgeon boats in 2008–2009.

### Use of Caspian Seal Products and Illegal Seal Hunting

Fishermen reported setting sturgeon nets to catch seals intentionally or by using clubs on ice and islands in spring time. We also recorded 9 reports of deliberate killing of moulted seals by Dagestani fishermen in 2008–2009. One instance of unofficial hunting (i.e. without licenses) was reported, when 200 seals were taken by fishermen on the ice in early spring 2009. Fishermen reported using seal skins (39 reports) and seal blubber (11 reports) from by-caught or deliberately killed seals. Skins were primarily taken in Dagestan while Kazakhstan fishermen reported using only blubber (Table 1).

### Evidence of by-catch among Stranded Seals

A total of 312 mostly highly decomposed carcasses were found during the May 2009 mass-stranding (218 - around Bautino and 94 - on Kulaly Island). The state of decomposition of most carcasses suggested a time of death 2 to 3 months prior to discovery. Of the 312 carcasses, 30 (9.6%) were found to be entangled in the remains of nets with mesh size 130–150 mm, which is typical of sturgeon nets. The state of decomposition did not allow any further assessment of cause of death for other carcasses.

## Discussion

The documented minimum by-catch from interview reports in 2008–2009 was 1,215 seals. This represents about 1.2% of the total Caspian seal population, estimated at ∼104,000 in 2005 [10], [11]. The Russian interview sample can be considered geographically representative. Sampling in Kazakhstan was more limited, but did include the main fishing settlements of the Atyrau region, the only populous area of the northern Kazakh coast. Sampling of the rest of the Kazakh coast and Turkmenistan was opportunistic. While in principal the interview sample size would be sufficient to allow fleet-wide extrapolation for the north Caspian Sea (from example via bootstrapping), it would not be appropriate due to the incomplete geographic scope in Kazakhstan, the opportunistic nature of interviews, and uncertainty over the proportion of total fishing effort in the north Caspian Sea represented by the interviews. Strukova and Guchgeldiyev [13] cite 2,130 illegal boats operating in Russia in 2007, but local representatives of the Border Guard Service and Federal Fisheries Agencies in Russia and Kazakhstan reported that this had fallen to 400 by the year of our survey due to stricter fisheries rules, decreasing sturgeon abundance, increased risks to personal safety and higher operating costs for fishing. Given that we report here a *minimum* by-catch rate derived from just 31 distinct sturgeon boats in 2008–2009 the true by-catch in the north Caspian Sea will be several times this minimum value, and potentially an order of magnitude greater. Fisheries-related mortality is also likely to be common in Azerbaijan, Iran, and areas of Turkmenistan and Kazakhstan not covered by this survey, so the total Caspian-wide fisheries related seal mortality will be greater still. The annual minimum by-catch mortality could account from 5 to 19% of annual pup production which has ranged between 6,250 and 25,100 for 2005–2011 as estimated from aerial surveys [11], [19].

Fishermen may have either avoided participating in interviews, or under-reported catch rates, both of which will lead to more conservative estimates of by-catch rate. Other informants could have given answers they believe an interviewer wanted to hear, or recalled rare events rather than common ones. These are issues many interview based studies have to deal with [8], and our protocol incorporated standard measures to minimize bias as far as possible. Given the limitations imposed by the illegal nature of the fishery, we focused on estimating a minimum documented by-catch as the most conservative approach to gain useful quantitative data from the participants. This can show whether the scale of by-catch merits concern from policy makers, and prioritize areas for further research. We were able to assess the likely scale of by-catch, and to identify potential high risk gear/location/season combinations, which can be used to guide interventions. This approach may prove useful in estimating the minimum scale of by-catch in other small-scale unregulated or illegal fisheries which are otherwise hard to assess. Extending interview effort and covering additional areas of coastline around the Caspian Sea could enable the adoption of stratified or truly randomized sampling. This should allow extrapolation of fleet-wide estimates and improve the robustness of comparative assessments of by-catch in the Caspian Sea.

Additional evidence of high by-catch levels comes from the examination of stranded carcasses. In May 2009 approximately 10% of 312 stranded carcasses showed direct evidence of entanglement in large mesh nets. As most of the carcasses were highly decomposed this should be treated as a minimum estimate, since the decomposition may obscure evidence of entanglement in other carcasses. This provides important physical support for the conclusion of high mortality rates due to by-catch from the interview study.

By-catch levels suggested by this study are comparable in scale to those which have caused critical threats to other endangered pinnipeds and small cetaceans [20], [21]. Conflict between seals and commercial fisheries generates high mortality in land-locked Ladoga and Saimaa ringed seal populations (*Pusa hispida*) [6], [20], [22]. An ecological risk analysis of the Saimaa ringed seal demonstrated that by-catch in combination with water level changes could drive the population to extinction [20].

High levels of by-catch have likely been occurring since the collapse of the Soviet Union in the early 1990s. The illegal Caspian sturgeon take in Russia is estimated at 1000–2000% higher than the official catch [13]. The actual total seal by-catch may therefore be of the order of greater than 10^5^ seals since the early 1990s. Such high rates of by-catch could imply catastrophic rates of decline, greater than the 3–4% per year estimated by Härkönen et al. [10], [11]. However, the rate of decline will be influenced by the age-structure of the mortality. For life-history models typical for small phocids, high rates of mortality among immature individuals may not cause catastrophic collapses, if mortality of fertile adult females is low [10]. Anecdotal comments from fishermen suggest by-catch is biased towards immature individuals, but the age structure among by-caught seals remains to be accurately quantified.

Large-scale official commercial hunting of Caspian seals ceased in the mid-1990s as it was considered economically unviable. The Caspian Commission on Aquatic Bioresources (an intergovernmental quango) currently sets a total quota of 18,000 seals annually across all Caspian countries. Russia is the only nation to actively take up its allocation at this time, and Kazakhstan has not issued licenses since 2006. Sporadic commercial hunts by Dagestanian teams have been carried out legally under an official annual quota up to 8,000 seals allocated to Russia since 2004 [23], with up to 4,600 seals taken in the most successful years. The mortality from hunting and by-catch can be put into context by considering the Potential Biological Removal (PBR) [24]. A sustainable-harvest mortality level for Caspian seals can be estimated from PBR = N min×0.5R max×RF [24], using the 2005 minimum population estimate (N min = 104,000; [10], [11], maximum net productivity rate (R max) of 0.12 (a typical value for small pinnipeds [24] and recovery factor (RF) value of 0.5 [24]. This yields 3187 animals a year, which is much less than current hunting quotas. The total annual by-catch rate may exceed this value several times over given that our survey covered only a small sample of total fishing effort. Therefore fisheries by-catch is suggested as being a key driver of population decline since the 1990s, and may currently be one of the most important threats to the species. The ongoing sporadic legal hunt will be an additional contributing factor to population decline. Modeling of the future demography of the population under different mortality scenarios is beyond the scope of this study, and is potentially limited by lack of detailed information on the age structure of mortality and the overall population.

Our interview data suggest Dagestanian fishermen often supply skins from by-caught seals to seal skin-processing businesses, which operate parallel to a legitimate factory for skins taken under license. Skins are used for making fur hats and coats which are presumably sold throughout Russia. The price for a freshly moulted pup (sivar) skin may reach US$100 in Dagestan. In Kazakhstan and Turkmenistan, seal blubber is used as a health tonic similar to cod liver oil, for crayfish bait and cattle feed. Seal oil sells for US$14.00 per liter in Turkmenistan markets (personal observations by the authors). More research is required to understand the full extent, trading pathways and economic value of the market for seal products, and its importance within the region.

Mitigation measures for Caspian seal by-catch will be closely allied to the solution for sturgeon poaching, since typical approaches to mitigating seal by-catch in legal fisheries elsewhere, e.g. observers, restrictions on time and location of fishing, modifications to gear will not be enforceable in an illegal fishery. Communities involved in poaching have limited options for other income due to poor economic opportunities along the coastal regions of the Caspian Sea [14]. Therefore solutions to poaching would need to include socio-economic changes, such as developing alternative livelihoods, alongside bolstering law enforcement, and reducing consumer demand for sturgeon products. The creation of properly resourced and enforced protected areas for the Caspian seal could reduce the risks of fisheries-related mortality. However, the social and political complexity of dealing with sturgeon poaching means that the prospects for eliminating by-catch and reducing impact on the seal population in the short term are likely to be low without sustained action from the Caspian governments.

## Supporting Information

File S1 **Supporting file containing Section S1 and Figure S1.** Section S1. Extended methods and questionnaire for fishermen interviews. Figure S1. Evidence of mass entanglement of Caspian seal groups. More than 20 Caspian seal carcasses entangled in a single sturgeon net (Photo © Brian Deacon, KBR-I&M, Leatherhead, UK, used with permission under a Creative Commons License).
(DOCX)
