# Supplementary material for: Assessment of Caspian Seal By-Catch in an Illegal Fishery Using an Interview-Based Approach
Source: PLoS One. 2013 Jun 26;8(6):e67074. doi: 10.1371/journal.pone.0067074 (PMC3694144; doi:10.1371/journal.pone.0067074)

**Supplementary Information**

**Section S1: Interview protocol**

Four trained native Russian or Kazakh speaking interviewers collected the data using a standardized interview protocol based on a questionnaire developed for similar research on Ladoga ringed seals [1]. Fishermen were approached by one or two researchers at local harbors, on the shore or in public places (markets, shops, cafes etc) with opening statements emphasizing the absence of any danger (e.g. no connection with authorities, anonymity of interviewees), the academic purpose of the visit, and the sincere interest in and importance of the information participants could contribute. Conducting the introductory stage of interviews in a friendly conversational manner was essential for further cooperation. If the fisherman was willing to talk, the interview continued with the protocol questions embedded in the natural conversation. Questionnaire design and interview techniques are very important when interviewing people involved in illegal activities. If the respondent trusts the interviewer, significant bias due to false or misleading answers can be avoided [2]. Enhanced item wording was often used to facilitate responding to sensitive questions and minimize question threat [3]. Where possible our interview included alternately phrased repeated questions to check consistency of answers. Sensitive questions (e.g. number of seals by-caught, characteristics of fishing gear etc.) were asked at the end of the conversation. A neutral attitude was expressed by interviewer in order to minimize influence on responses. Some fishermen volunteered freely, showing their own interest and asking questions about the seal research in general. Other individuals would only talk openly when the researchers were introduced by their friends or colleagues. Therefore in some cases, prior local contacts gained in previous reconnaissance visits were useful for obtaining the data. Perceived low risk of imprisonment or incurring significant fines may partly account for why individuals were not afraid to talk openly about illegal activity they were personally involved in. A dictaphone was used to record all the conversations. Interviews were transcribed and the data summarized in a spreadsheet by tabulating name of settlement, year of activity, season of activity, fishing area, target fish species, type of gear, cases of fish and fishing gear damaged by seals, length of nets in a set, number of nets in set, mesh size, number of seals by-caught per net per boat, plus notes on seal skin or blubber use and any additional seal hunting activity.

**Interview questions for assessing Caspian seal by-catch**

Based on a questionnaire developed for by-catch research on Ladoga ringed seals [1].

- Fishing area
- Type of fishing gear. Mesh size. Depth of fishing gear setting
- Type of fish
- Quantity of gear (or length of nets).
- Season or period of fishing
- Do you meet seals in your fishing area?
- Do seals damage fish in your gear? Type of the fish? Season?
- Do seals damage your gear? Type of the gear?
- Are there any seals entangled in your fishing gear? Type of gear and season?
- How many seals are by-caught in your fishing gear during a year/season/one set?
- What do you do with seals by-caught?
- Do you or your colleagues use any seal products?
- Have you or your colleagues ever killed seals deliberately?
- Do you think that seals are detrimental to fisheries?
- Your personal attitude to seals

“______” ___________ 2009.

**References**

1. Verevkin, M., Trukhanova, I., and T. Sipilä. 2008. The relationship of seals and fisheries in the Leningrad region. Proceedings of Fifth International Conference, Marine mammals of the Holarctic, Odessa, 562-565. Published by the Marine Mammal Council of the Russian Academy of Sciences, Moscow, Russia.
2. [Gavin, M. C., Solomon, J. N., and S. G. Blank. 2010. Measuring and monitoring the illegal use of natural resources. Conservation Biology 24: 89-100](http://onlinelibrary.wiley.com.oca.ucsc.edu/doi/10.1111/j.1523-1739.2009.01387.x/pdf).
3. Catania, J. A., Binson, D., Canchola, J., Pollack, L. M., Hauck, W., Coates, T. J., 1996. Effects of interviewer gender, interviewer choice, and item context on responses to questions concerning sexual behavior. Public Opinion Quarterly 60: 345-375.

**Figure S1.**

Evidence of mass entanglement of Caspian seal groups. More than 20 Caspian seal carcasses entangled in a single sturgeon net (Photo © Brian Deacon, KBR-I&M, Leatherhead, UK, used with permission under a Creative Commons License).


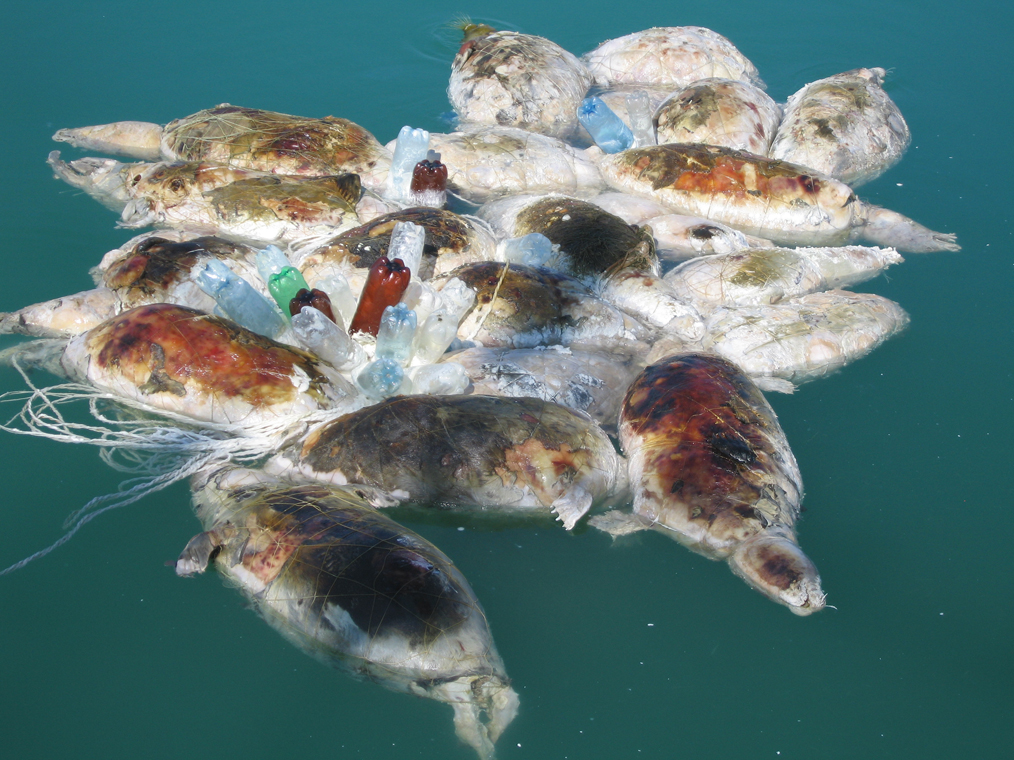

Supplement: File S1 — Supporting file containing Section S1 and Figure S1. Section S1. Extended methods and questionnaire for fishermen interviews. Figure S1. Evidence of mass entanglement of Caspian seal groups. More than 20 Caspian seal carcasses entangled in a single sturgeon net (Photo © Brian Deacon, KBR-I&M, Leatherhead, UK, used with permission under a Creative Commons License). (DOCX) [file pone.0067074.s001.docx]
